# Factors Influencing Nurses’ Decisions to Leave or Remain in the Home and Community Care Sector During the COVID-19 Pandemic

**DOI:** 10.3390/healthcare12222212

**Published:** 2024-11-06

**Authors:** Denise M. Connelly, Nicole A. Guitar, Travis A. Van Belle, Sandra M. McKay, Emily C. King

**Affiliations:** 1 School of Physical Therapy, Western University, 1201 Western Rd, London, ON N6G 1H1, Canada; nguitar@uwo.ca; 2 VHA Home HealthCare, 30 Soudan Avenue, Suite 600, Toronto, ON M4S 1V6, Canada; tvanbelle@vha.ca (T.A.V.B.); smckay@vha.ca (S.M.M.); emily.king@vha.ca (E.C.K.); 3 Dalla Lana School of Public Health, University of Toronto, 155 College Street, Room 500, Toronto, ON M5T 3M7, Canada; 4 Centre for Research Expertise in Occupational Disease, 223 College Street, Toronto, ON M5T 1R4, Canada; 5 Institute for Health Policy, Management, and Evaluation, University of Toronto, 155 College Street, Suite 425, Toronto, ON M5T 3M6, Canada; 6 Department of Physical Therapy, University of Toronto, 500 University Avenue, Toronto, ON M5G 1V7, Canada; 7 Ted Rogers School of Management, Toronto Metropolitan University, 55 Dundas Street West, Toronto, ON M5G 2C3, Canada; 8 The Institute for Education Research (TIER), University Health Network, 222 St. Patrick Street, Toronto, ON M5T 1V4, Canada; 9 Michener Institute of Education, University Health Network, 222 St. Patrick Street, Toronto, ON M5T 1V4, Canada; 10 Micheal Garron Hospital, Toronto East Health Network, 825 Coxwell Avenue, East York, ON M4C 3E7, Canada

**Keywords:** home care, community care, nurses, retention, COVID-19, survey, emotional intelligence, burnout

## Abstract

**Background/Objectives:** Home and community care (HCC) nurses experienced increased occupational challenges during the COVID-19 pandemic, including increased workloads, job stressors, and occupational risks, like virus exposure. The objective of this study was to elucidate what factors influenced nurses’ decisions to stay in their role, take a temporary leave, or exit HCC during the COVID-19 pandemic. **Methods:** A secondary analysis of data collected using a cross-sectional online open survey distributed among HCC Registered Practical Nurses across Ontario between June and September 2022 was conducted. The factors contributing to nurses’ decision to remain in HCC, temporarily leave, or exit the sector were evaluated using multinomial logistic regression (*p* < 0.05). **Results:** Of the 664 participants, 54% (*n* = 357) stayed in the HCC sector, 30% (*n* = 199) temporarily left, and 16% (*n* = 108) exited the sector. Nurses with greater years of experience working in HCC and those who avoided infection were more likely to stay in their role in HCC, which may reflect strong relationships with long-term clients, opportunity and accumulated experience to increase income, and maintenance of good health. Nurses with higher levels of emotional intelligence were more likely to take leaves and exit HCC, suggesting that stepping away may have been a strategy to safeguard themselves. **Conclusions:** HCC leadership should prioritize the development of solutions to support nurses in the HCC workforce, including those with fewer years of experience. This may promote nurses’ participation in the sector, particularly during times of heightened occupational challenges and crises, like COVID-19.

## 1. Introduction

Nurses working in home and community care (HCC) provide care to people of all ages in their home, at school, or in the community [1]. Like their peers in other sectors, nurses working in the HCC sector during the Coronavirus Disease 2019 Pandemic (COVID-19) faced multiple increased occupational challenges across the globe, including staffing shortages, increased caseloads, high virus exposure risk, and, in some cases, inequities regarding access to resources and supports, such as personal protective equipment and infection prevention training [2]. These nurses reported feeling disrespected, frustrated, overwhelmed, and burned out during the pandemic [3,4]. As with other healthcare sectors, recent reports indicate that nurses are leaving the HCC sector en masse internationally [5], causing a “crisis in the nursing labour market” [6] (p. 1). Further, during COVID-19, nurses were the most frequently infected healthcare personnel, according to the results of a systematic review and meta-analysis of COVID-19 infection in healthcare workers [7]. In the wake of COVID-19, “many [HCC nurses] who previously enjoyed their nursing role [were] re-evaluating their relationship to their careers; for some, this meant stronger professional attachment and for others, it meant intentions to leave [the sector]” [8] (p. 10). It is not currently known what factors influenced nurses’ decisions to continue working in the HCC sector, take a temporary leave, or exit the sector during COVID-19.

HCC nurses report that the ‘autonomy’ associated with working in HCC is an attractive aspect of working in the sector. This includes “autonomy over decision-making about care, freedom in work scheduling, and working in a self-directed team” [9] (p. e94). Balanced against this positive aspect of HCC work, there are multiple factors that make work within HCC challenging, including but not limited to the following: having to adapt to changing work environments and heavy workloads, managing occupational stress regarding infection transmission and safety at work, constantly evolving protocols, staffing shortages, and social isolation [10,11,12,13,14,15,16,17]. Within this context, the factors associated with nurses’ intentions to remain working in HCC include the following: increasing years of age, treating a greater variety of clients, experiencing greater meaningfulness of work, having greater income stability, greater continuity of client care, experiencing more positive relationships with supervisors, experiencing better work–life balance, and being more satisfied with their salary and benefits [18].

A recent scoping review found some limited research about how emotional intelligence (EI) relates to the retention of nurses [19], but an integrative review from 2019 concluded that EI concepts are “central to nursing practice” and that emotionally intelligent leaders in nursing practice promote employee retention, quality of client care, and client outcomes [20] (p. 1624). EI is defined as “an individual’s capacity for recognizing their own feelings and the feelings of others, and the process of regulating feelings and expressions in response to situations” [21] (p. 396). Research suggests that EI correlates with organizational outcomes, like individual performance, employee retention, team effectiveness, and collaboration [22,23,24]. In research with nursing students specifically, there is evidence that EI relates positively to the retention of clinical staff nurses [25] and to the nurses’ intentions to remain [26]. It is not currently known how EI relates to HCC nurse retention specifically during a healthcare crisis, like the COVID-19 pandemic. 

Previous research suggests that the less ‘embedded’ nurses are within their organizations, the more likely they are to leave their roles [27]. ‘Embeddedness’ consists of “three sets of influences on employee retention: fit (the extent to which an employee’s job and community are similar to or fit with the other aspects of the employee’s life); links (the strength of the employees connections to other people or activities); [and] sacrifice (the ease with which links can be broken, such as what benefits and advantages employees would give up if they left the organization)” [27] (p. 469). Previous work by Nizzer et al. suggests that challenging work conditions impacted and continue to impact each of these elements of embeddedness, with potential implications for nurse retention during COVID-19 [8]. Nurses who are more ‘embedded’ may have more work experience, as ‘embedding’ may happen over time. Being more ‘embedded’ within their organization may also reduce social isolation experienced by nurses working in HCC, which has long been identified as an issue for people working within HCC [8,28,29,30]. The degree to which embeddedness impacts HCC nurse retention during a healthcare crisis like COVID-19 has not been fully explored.

To inform ongoing recruitment and retention efforts for this nursing workforce, this research aimed to understand what factors influenced nurses’ decisions to stay in, temporarily leave, or exit the HCC sector during the COVID-19 pandemic. 

## 2. Materials and Methods

### 2.1. Study Design and Participants

A secondary analysis was conducted using cross-sectional survey data first reported in Connelly et al. [31], which presents initial findings from a convenience sample of Registered Practical Nurses in Ontario who worked in home and community care (HCC) between January 2020 and September 2022. An open electronic survey was distributed through the Registered Practical Nurses Association of Ontario (WeRPN) to Registered Practical Nurses in Ontario who worked in home and community care (HCC) between January 2020 and September 2022, as per Dillman’s 2011 tailored design method [32]. Ethics approval for the study was provided by the Research Ethics Board at Western University. For additional details of the study design and participants, refer to Connelly et al. [31]. The Checklist for Reporting Results of Internet E-Surveys (CHERRIES; Eysenbach [33]) was used (see Supplementary material of Connelly et al. [31]).

### 2.2. Survey Design 

The survey consisted of 7 sections: screening and consent, where informed consent was obtained from all subjects involved in the study (3 items); Connor Davidson Resilience Scale (CD-RISC-10; 10 items with a cut-off score of ≥80 that can be used to indicate the presence of personal resilience); Resilience at Work (R@W) Scale (20 items that measure a person’s personal resilience in the workplace through seven domains of resilience); Wong and Law Emotional Intelligence Scale (WLEIS) for Nurses (16 items specific to measuring self-reported capacity to examine and control emotions); Registered Practical Nurse (RPN) Experience (10 items); questions related to the COVID-19 pandemic (46 items); and questions relating to Social Determinants of Health and Demographics (15 items). Connor and Davidson (2003) have reported the Cronbach’s alpha of the CD-RISC-25 scale to be 0.89, with a reliability coefficient of 0.87 reported for this scale through test-retest reliability within a four-week interval [34]. The CD-RISC-10 demonstrated satisfactory reliability (Cronbach’s alpha = 0.81) in student nurses and satisfactory validity with significant correlations with outcomes of self-esteem, depression, and psychological distress [35]. The R@W Scale does not have known psychometric properties; however, this scale has been used to assess the resilience of nurses at work in a variety of settings, which enables comparison [36,37,38]. The internal consistency of the WLEIS is high (i.e., Cronbach’s alpha ranges from 0.83–0.90) [39]. The WLEIS also has acceptable reliability as well as convergent and discriminant validity in healthcare professionals specifically [40]. Survey items broadly consisted of two types of questions: categorical items were binary (e.g., yes/no) or multiple-choice, while ordinal items were measured using Likert scales (e.g., a 5-point Likert scale). 

### 2.3. Demographics and Personal Experience Variables

The survey included various demographic items, including gender, ethnicity, age, marital status, citizenship status, income, physical health status, and mental health status. To support transparency while respecting privacy, analysis combined gender identities such that a “gender-diverse” identity includes those who identified as Two-Spirit, Non-binary, Gender non-conforming, Genderqueer, or Third gender. Subsequently, for the regression analysis, gender-diverse individuals and those who preferred not to answer were combined with women to permit their inclusion despite low sample size. Similarly, ethnic identities were collapsed based on sample size to support regression analysis. Past physical and mental health status (e.g., “How would you rate your mental health prior to COVID-19”) were combined into a single, overall past health status variable. 

The impact of the pandemic on various aspects of life, including household responsibilities, parenting, caregiving, relationships, personal hygiene, and professional network, was assessed using a 5-point Likert agreement scale (0—strongly disagree to 4—strongly agree). Parenting and caregiving responsibilities (e.g., “COVID-19 made it more difficult to work while maintaining my caregiving responsibilities”) were combined into a single variable to capture familial caregiving.

### 2.4. Work-Related Variables

Participants were asked to share the number of years they had been registered as a nurse, number of years working in HCC, and number of weekly hours worked. Additional categorical items included their employment status, whether they worked in other sectors, whether they had ever tested positive for COVID-19, whether their role included formal supervision of others, and identification of the geographies in which they worked (subsequently collapsed into the larger Ontario Health administrative regions of West, Central, Toronto, East, and North [combining North East and North West]). 

A 5-point Likert agreement scale ranging from 0—strongly disagree to 4—strongly agree was used to collect to what degree participants agreed with statements that their caseload increased, caseload fluctuated, travel requirements changed, virtual appointments increased, non-client-facing hours increased, referrals increased, and severity of clients’ care needs increased. The agreement scale was also used to assess the level to which individuals agreed with statements about being part of a team, that their employer cared about their wellbeing, and that they had opportunities to connect with their supervisor and opportunities to connect with their colleagues (e.g., “To what extent do you agree that your employer(s) provides opportunities for you to connect with your colleagues”). A 5-point Likert satisfaction scale (0—extremely dissatisfied to 4—extremely satisfied) was used to measure participants’ satisfaction with their income, professional development opportunities, educational opportunities, workplace recognition, Employee Assistance Programs (EAP), and employer benefits (e.g., “Please indicate how satisfied you have been with the following based on your home and community care RPN employment during COVID-19: Your Employee benefits”).

Nurses were also asked to rate their satisfaction with access to personal protective equipment (PPE), quality of PPE, quality of Infection Prevention And Control (IPAC) training, access to IPAC support, and their ability to access COVID-19-related information from their employer and their regulatory body (College of Nurses of Ontario). They also shared how satisfied they were with their connection with their supervisor and their employer, communication with their supervisor about COVID-19, and communication received from their employer about COVID-19 pandemic protocols (e.g., “In your work as a home and community care RPN during COVID-19, how satisfied have you been with the communication you received from your employer about the COVID-19 pandemic protocols and procedures”). Participants were asked whether they agreed that their direct supervisors’ and employer(s)’ communication was clear, concise, timely, and relevant (e.g., “During COVID-19, I believe the communication I received from my employer(s) in home and community care was relevant”). These aspects of communication were combined across supervisor and employer(s) to form clear, concise, timely, and relevant communication variables, respectively. 

### 2.5. Assessment Scales

Three assessment scales, including the 10-item Connor-Davidson Resilience Scale (CD-RISC-10) [34], the Resilience at Work Scale^®^ (R@W) [41], and the Wong and Law Emotional Intelligence Scale (WLEIS), were used [39]. The CD-RISC-10 assesses resilience concepts using 10 items, each rated on a 5-point Likert scale ranging from ‘not true at all’ (0) to ‘true nearly all the time’ (4), with total scores ranging from 0 to 40, and higher scores indicating greater personal resilience [42]. The R@W scale includes 20 items to measure resilience within workplace settings, each rated on a 7-point Likert scale ranging from ‘strongly disagree’ (0) to ‘strongly agree’ (6), where higher scores are indicative of higher resilience. Finally, the WLEIS assesses nurses’ emotional intelligence using 16 items, each measured using a 7-point Likert scale ranging from ‘strongly disagree’ (1) to ‘strongly agree’ (7), where higher scores indicate higher EI. Further description of these scales and selection rationale is provided in Connelly et al. [31].

### 2.6. Decision to Work in HCC

The outcome of interest in this secondary analysis was nurses’ decision to stay, temporarily leave, or exit HCC during the COVID-19 pandemic. This was assessed across three items. The first item asked whether a leave of absence was taken, with an opportunity to provide additional details regarding reasons for leaving, if applicable. Two items asked whether participants considered exiting HCC, and if they considered leaving, their decision (yes, no, temporarily). For those who exited, additional details could be provided regarding their motivation for exiting. 

### 2.7. Analysis and Variable Selection

Since ordinal/numeric data were non-normally distributed, bivariate tests were completed using the Kruskal–Wallis Rank Sum Test. Chi-square and Fisher’s exact tests were used for categorical data, as appropriate. Multinomial logistic regression was used to assess the relative association between variables and nurses’ decisions to stay, temporarily leave, or exit HCC. Findings were considered significant if *p* < 0.05. Relative risk ratios were computed to support interpretation of results; risk ratios with confidence intervals that did not include 1 were considered significant. 

Bivariate analysis highlighted many significant associations between independent variables and nurses’ decision to stay, temporarily leave, or exit HCC. The inclusion of non-demographic and employment characteristic variables in the regression model was guided by a combination of theory and exploratory factor analysis. Factor groupings were assembled to identify those with similar impacts. Mathematically identified factors were further divided according to the overarching theoretical constructs that the included variables represented. Variables were included, individually or in combination, in ways that respected these groupings and improved the model fit, as assessed using the Akaike Information Criterion (AIC) [43]. Variables with a strong theoretical basis for inclusion (e.g., demographics) were included regardless. The model was then checked by removing each included variable individually to confirm whether its inclusion improved AIC. Previously excluded variables were re-added individually to assess their impact on the model and confirm that their exclusion improved model fit. The presence of multicollinearity between variables was also assessed using Generalized Variance Inflation Factor (GVIF) [44]. All analyses were performed using RStudio (R version: 4.3.2, Posit, PBC). 

## 3. Results

### 3.1. Sample

Of the 724 valid survey submissions received, 664 were fully completed (i.e., they contained no missing data). Participants were quite evenly split between women (49.4%) and men (49.5%), with 1.1% identifying as gender-diverse/prefer not to say. Individuals predominantly identified as Black (13.7%), East/Southeast Asian (5.7%), South Asian (11.4%), and White (59%), with the remaining 10.1% representing other identities. Respondents were largely Canadian citizens (45.5%) or permanent residents (44.3%), with the remaining participants identifying as temporary residents (9.9%) and ‘other’ (0.3%). Participants averaged 38 (±8.07) years of age, 13.2 (±5.85) years of nursing experience, and 6.8 (±4.54) years of experience working in HCC (Table 1).

### 3.2. Decision to Continue Working in HCC

Among the participants, approximately half the respondents (53.8%) continued to work in HCC during COVID-19, 30.0% chose to temporarily leave, and the remaining 16.3% exited (i.e., left their role working in HCC). 

### 3.3. Variables Influencing Both Temporary Leaves and Sector Exits

Multiple individual-level variables were significantly associated with both temporary leaves and exits from HCC during the COVID-19 pandemic (Table 2 and Table 3). When controlling for other variables, higher emotional intelligence scores were significantly associated with temporary leaves (RRR: 1.04 CI: [1.01, 1.07]) and exits from the sector (1.10 [1.06, 1.14]). Similarly, participants who reported an increase in household income were 2.4 times (2.44 [1.27, 4.69]) and 4.5 times (4.52 [2.04, 10.02]) more likely to temporarily leave and exit the sector, respectively. Those who received COVID-19-related, governmental, supplementary income were about 1.9 times (1.88 [1.15, 3.08]) more likely to temporarily leave and 2.4 times (2.35 [1.23, 4.51]) more likely to exit the sector. Lastly, those who reported having tested positive for COVID-19 were over 4 times (4.43 [2.53, 7.77]) and 3.5 times more likely (3.55 [1.83, 6.89]) to temporarily leave and exit, respectively. 

Among work-related variables, experience in HCC was associated with staying in HCC; each additional year of experience reduced the likelihood of temporarily leaving or exiting the sector by more than 10% (temporary leave: 0.89 [0.83, 0.96]; exit: 0.88 [0.80, 0.97]). Conversely, individuals who worked in the Toronto region were over 2 times (2.26 [1.06, 4.81]) and 4.5 times (4.63 [1.87, 11.45]) more likely to take a temporary leave and exit, respectively. Those with higher satisfaction with their employer’s communication regarding COVID-19 protocols were also more likely to temporarily leave and exit the sector (temporary leave: 1.30 [1.02, 1.66]; exit: 1.48 [1.10, 1.99]). 

### 3.4. Variables Influencing Only Temporary Leaves

Some variables were significantly associated with leaves but did not impact exits (Table 3). At the individual level, those with a decrease in household income or reported pandemic impacts on their relationships with friends were nearly 2.5 times (2.45 [1.27, 4.74]) and 1.3 times (1.25 [1.02, 1.54]) more likely to temporarily leave, respectively. Finally, individuals with ‘other’ ethnic identities (Indigenous–First Nations, Latin American, Middle Eastern, and Other) beyond the largest four groupings were over 2 times (2.25 [1.07, 4.70]) more likely to temporarily leave HCC. 

At work, those who entered the pandemic already in supervisory roles were 2.8 times (2.80 [1.23, 6.36]) more likely to temporarily leave but not exit. Higher rates of leaves were found for participants who reported higher satisfaction with benefits and more concise communication (1.53 [1.18, 1.99] and 1.56 [1.08, 2.24]), respectively. Conversely, relevant communication and increased weekly hours of work were associated with staying in HCC (relevant communication: 0.55 [0.37, 0.80]; weekly hours: 0.97 [0.95, 0.998]). 

### 3.5. Variables Influencing Only Sector Exits 

Some individual variables were significantly associated with sector exits but not temporary leaves (Table 3). Compared to those identifying as White, participants who identified as South Asian were almost 3 times more likely to exit the sector (2.90 [1.14, 7.35]), while Black individuals were 60% less likely to exit (0.40 [0.16, 0.99]). Individuals who were separated/divorced were 3 times (3.08 [1.37, 6.93]) more likely to exit the sector. Those who reported better pre-pandemic health were more likely to stay (0.64 [0.45, 0.91]).

Some work-related variables were also associated with staying rather than exiting HCC. Those with an increased caseload were about 30% less likely to exit (0.73 [0.57, 0.94]). Participants who reported concise communication, timely communication, and satisfaction with their supervisor’s communication were 43% (0.57 [0.37, 0.89]), 39% (0.61 [0.40, 0.92]), and 30% (0.70 [0.52, 0.94]) less likely to exit, respectively. Lastly, those who reported greater opportunities to connect with colleagues were 30% less likely to exit (0.70 [0.52, 0.95]).

## 4. Discussion

This study sought to understand the factors that influenced nurses’ decisions to continue working in the HCC sector, take a temporary leave, or exit the sector during the COVID-19 pandemic. Data were collected from RPNs working in HCC across Ontario using a cross-sectional open online survey. The factors contributing to nurses’ decisions to remain in HCC, temporarily leave, or exit the sector were evaluated using multinomial logistic regression. More than half of the participating RPNs stayed in their roles. Understanding what factors were associated with nurses’ decisions to take temporary leaves or exit the sector can inform future retention efforts by providing insight into factors that may have contributed to nurses’ decisions to continue to work in HCC during COVID-19.

In this study, higher scores on EI, as measured by the WLEIS, were significantly associated with both taking a temporary leave and exiting HCC. EI has been thought to “buffer the effects of negative emotions on job burnout in nurses” [45] (p. 1). During COVID-19, direct-care staff, including nurses, repeatedly reported experiencing increased burnout [46,47], which results from “prolonged exposure to high job demands in the absence of enough resources to compensate for their effects” [45] (p. 1) [48,49,50]. In the present study, those with higher EI were more likely to step away from HCC during COVID-19, perhaps to “buffer the effects of negative emotions” in their work during the pandemic [45] (p. 1). That is, stepping away from their work may have been a strategy that facilitated their safeguarding from the “negative” aspects of their work. Additionally, nurses who reported also having a supervisory role had a higher rate of leaves when compared to nurses who did not have a supervisory role. This suggests that nurse supervisors may have had a greater need to “buffer the negative emotions” [45] (p. 1) during the pandemic because of the changes in their workloads to provide support for their staff members who experienced unprecedented levels of personal and professional challenges during COVID-19 [51].

Having a change in household income (either increase or decrease), compared to pre-pandemic levels, or having fewer work hours were both factors that were associated with nurses taking a temporary leave from the sector. The nurses’ satisfaction with benefits was also associated with taking a leave. It may be the case that nurses with benefits that allowed them to afford taking a leave from their work were more likely to do so. The findings related to changes in household income highlight that there are factors not captured in this study that would influence household income (e.g., changes in the incomes of other people within their households), which may also influence a nurses’ choice to take a leave from work. With respect to the nurses’ incomes, nurses who previously held two or more jobs (approximately 20% of RPNs) [52] were forced by provincial policy to choose a single employer, which could impact both hours of work and overall income. Ontario’s ‘one-employer’ policy aimed to reduce the potential spread of the virus and impacted the RPNs included in this study but was not unique to this provincial jurisdiction [53]. Other provinces and countries implemented similar policies during COVID-19 [54].

The autonomy of HCC nurses, reported as a positive work-related factor prior to COVID-19, may have been experienced as isolation during the pandemic. This includes “autonomy over decision-making about care, freedom in work scheduling and working in a self-directed team” [9] (p. e94). In the present study, a collection of factors, such as increased caseload, the pandemic’s impact on nurses’ professional network and friendships, and whether nurses had opportunities to connect with their colleagues, suggest that during COVID-19, ‘autonomy’ and ‘flexibility’ may have shifted to feel more like ‘isolation’ on the job. Globally, social isolation has been routinely identified as an issue for people working within HCC, even prior to the COVID-19 pandemic [8,28,29,30]. International evidence suggests that COVID-19 exacerbated the nurses’ sense of professional isolation [28,55,56]. Nurses within HCC “felt alone” during COVID-19 while “simultaneously shouldering a significant responsibility for clients facing a novel and unfamiliar illness” [56] (p. 321).

This shift may have impacted the degree of ‘embeddedness’ experienced by HCC nurses, as the characteristics of HCC that made it a desirable place to work for nurses pre-pandemic became disadvantages when navigating through a health sector during a global crisis. ‘Embeddedness’ consists of “three sets of influences on employee retention: fit (the extent to which an employee’s job and community are similar to or fit with the other aspects of the employee’s life); links (the strength of the employees connections to other people or activities; [and] sacrifice (the ease with which links can be broken, such as what benefits and advantages employees would give up if they left the organization)” [27] (p. 469). Specifically, when it comes to how the characteristics of HCC that made it a desirable place to work for nurses pre-pandemic became disadvantages, ‘fit’ with other aspects of nurses’ lives may have been impacted by the erosion of autonomy through the removal of nurses’ choice about work location(s) and limitations placed upon nurses to have a single employer. Further, as the links that define the “strength of the employee’s connections to other people or activities” in the organization decreased in response to physical distancing and other public health measures and the ease with which the links could “be broken” increased [27] (p. 469), nurses found themselves less embedded within their organizations and more isolated by the pandemic-exacerbated conditions of their roles as HCC nurses.

Nurses’ considerations about their own personal safety may also have been associated with their decisions to stay in the sector, take a temporary leave, or exit. Specifically, testing positive for COVID-19 was significantly associated with both taking a temporary leave and exiting the sector in this study. In addition to the direct consequences of infection, including potentially temporary or long-term disability associated with the sequelae of COVID-19 infection, anxiety related to the potential transmission of COVID-19 to family has been documented as a significant stressor for many healthcare providers. Given this reality, it is unsurprising that COVID-19 infections impacted nurses’ choices regarding participation in the HCC sector [57]. In the absence of their personal health being at risk, people may have been more likely to continue working in HCC during COVID-19, and indeed we see that nurses who self-reported better past health than others were also more likely to stay working in the HCC sector. Feeling as though their work and exposure risk were impacting their relationships with family and friends was another factor significantly associated with RPNs taking a temporary leave from HCC during the pandemic. Ethnic identity was another factor identified as being related to nurses taking a leave or exiting the sector. The nurses’ reasons for exiting the sector in the current study were not captured; however, it is possible that the surge in anti-South Asian racism impacted some of their willingness to engage with the community in the way required of HCC nurses [58]. Having to move through the community to provide care as a nurse is inherently vulnerable regardless of ethnic identity, but the influence of racism may have impacted the perceptions of personal safety for particular groups, making them more likely to exit the sector. 

The degree of embeddedness within their organizations may also have been impacted by the nurses’ years of HCC work experience, strength of relationships with clients and colleagues, and the quality of communication with their employer—all of which were associated with staying in the sector. Respondents with greater years of experience took fewer temporary leaves and exited the sector less. Within home care, research suggests that “good nursing care is built on trusting relationships”, which starts with “establishing the relationship” and is maintained through ongoing “conscious efforts” with “reciprocity” between the nurse and the client as a requirement in the relationship [59] (p. 89). Consequently, newer nurses (i.e., nurses with fewer years of experience working in HCC) may not have had the time needed to develop relationships with their clients and their family members. In addition, they may have less stable work schedules, which would impact the ability to build strong bonds with the clients. Therefore, nurses with less experience may not have the same depth of relationships and therapeutic alliances with their clients as the more experienced nurses did, causing them to seek alternate employment in other sectors with greater resources and immediate supports. For those 54% of RPNs who remained in their work within the HCC sector, it appears that perhaps the relationships they held with their clients and their families were key motivators for work within HCC. Furthermore, greater opportunities to connect with colleagues and supervisors that promote stronger connections, consistent with Nizzer et al. [8], were also associated with nurses staying in their role. Working a greater number of hours, which would provide opportunities for increased connections with clients and their family members, colleagues, and supervisors, could increase a nurse’s embeddedness within their organization. Lastly, better communication from employers during the pandemic, which could also be seen to increase the embeddedness of a nurse within their organization, was also associated with nurses remaining in HCC. For those nurses who remained working within HCC in the present study, findings suggest that: nurses with greater levels of experience working in HCC, who subsequently may have had stronger relationships with their clients, colleagues, and supervisors, who received communication from their employers, and who worked greater numbers of hours may have been more likely to stay in their role in HCC. 

## 5. Limitations

This study analyzed cross-sectional data from a single point in time and captured experiences from the start of the pandemic until that date. It is not possible to determine for how long people who stayed working in HCC continued to work, if someone who exited may have returned, or if those who took temporary leaves returned or subsequently exited the sector. Convenience sampling was also used for this survey, with a broad provincial-wide recruitment strategy. As a result, our findings are not generalizable to all RPNs, nor to their similar roles internationally, such as Licensed Practical Nurses, Enrolled Nurses, and Associate Nurses. As this was an open online survey, we also cannot exclude the possibility of a bias in people with stronger (positive or negative) feelings choosing to participate or social desirability bias leading respondents to share misleading information to please the researchers. 

## 6. Conclusions

The survey findings point to the individual and work-related factors related to nurses’ decisions to remain in HCC, temporarily leave, or exit the sector during the COVID-19 pandemic. Clarity regarding these factors may inform HCC organizational-level efforts to support the nurse workforce and promote retention. The emotional intelligence of nurses and factors influencing their degree of ‘embeddedness’ within the organization appear to play substantial roles in retention. While many contextual factors are beyond an organization’s control, it is clear that the factors related directly to the strength of peer and supervisory connections must be areas of organizational focus. Through a continual focus on strengthening these relationships, organizations can ensure that they are less fragile when crises occur, and a heightened focus on this connectedness in times of crisis is likely to support retention. Further exploration of factors impacting the regulation of feelings and expressions in response to work situations (emotional intelligence), inherent in the practice of nursing, is needed to promote job satisfaction and nurse well-being to support their occupation of caring for others in the much-needed home and community health care sector. 

## Figures and Tables

**Table 1:** Sample characteristics.

Variable	Description	Proportion (%) or Mean (±SD)
n		664
Gender	Woman	328 (49.4%)
	Man	329 (49.5%)
	Gender-diverse/Prefer not to say	7 (1.1%)
Ethnicity	Black	91 (13.7%)
	East/Southeast Asian	38 (5.7%)
	South Asian	76 (11.4%)
	White	392 (59%)
	Other identities	67 (10.1%)
Age		37.99 (±8.07)
Marital Status	Single	64 (9.6%)
	Married/Common law	502 (75.6%)
	Separated/Divorced	81 (12.2%)
	Widowed	17 (2.6%)
Citizenship Status	Canadian citizen	302 (45.5%)
	Permanent resident	294 (44.3%)
	Temporary resident	66 (9.9%)
	Other	2 (0.3%)
Registered years as a nurse		13.22 (±5.85)
Years worked in HCC *		6.78 (±4.54)
Employment Status	Full-time in 1 or more roles	453 (68.2%)
	Part-time	211 (31.8%)
Ontario Health Region	West	231 (34.8%)
	Central	217 (32.7%)
	Toronto	75 (11.3%)
	East	105 (15.8%)
	North (East and West)	36 (5.4%)

Note: SD = Standard Deviation; HCC = home and community care. * regardless of employment status.

**Table 2:** (**A**). Bivariate associations between demographic and workplace characteristics and decision to stay in, temporarily leave, or exit from HCC. (**B**). Bivariate associations between experience and satisfaction variables (0–4 scale) and decision to stay in, temporarily leave, or exit HCC.

**(A)**					
**Variable**	**Description**	**Stay**	**Temporary Leave**	**Exit**	***p*** **-Value**
		**n (%) or ** **Median [IQR]**	**n (%) or ** **Median [IQR]**	**n (%) or ** **Median [IQR]**	
n		357 (53.8%)	199 (30.0%)	108 (16.3%)	
Gender	Woman and Gender-diverse/Prefer not to say	185 (51.8%)	100 (50.3%)	50 (46.3%)	0.601
	Man	172 (48.2%)	99 (49.7%)	58 (53.7%)	
Age		38.00 [30.00, 42.00]	40.00 [34.00, 45.00]	35.00 [31.00, 43.00]	**<0.001**
Ethnicity	Black	60 (16.8%)	19 (9.5%)	12 (11.1%)	**0.029**
	East/Southeast Asian	20 (5.6%)	10 (5.0%)	8 (7.4%)	
	South Asian	28 (7.8%)	29 (14.6%)	19 (17.6%)	
	White	211 (59.1%)	120 (60.3%)	61 (56.5%)	
	Other identities	38 (10.6%)	21 (10.6%)	8 (7.4%)	
Marital status	Single	44 (12.3%)	15 (7.5%)	5 (4.6%)	0.157
	Married/Common law	266 (74.5%)	152 (76.4%)	84 (77.8%)	
	Separated/Divorced	37 (10.4%)	28 (14.1%)	16 (14.8%)	
	Widowed	10 (2.8%)	4 (2.0%)	3 (2.8%)	
Citizenship status	Canadian citizen	157 (44.0%)	75 (37.7%)	70 (64.8%)	**<0.001**
	Permanent resident	155 (43.4%)	104 (52.3%)	35 (32.4%)	
	Temporary resident	43 (12.0%)	20 (10.1%)	3 (2.8%)	
	Other	2 (0.6%)	0 (0.0%)	0 (0.0%)	
Past health		3.00 [2.50, 3.50]	3.00 [2.00, 3.50]	3.00 [2.50, 3.50]	**0.014**
Test Covid-positive	No	260 (72.8%)	74 (37.2%)	52 (48.1%)	**<0.001**
	Yes	97 (27.2%)	125 (62.8%)	56 (51.9%)	
Connor-Davidson Resilience score (/40)		29.00 [24.00, 33.00]	31.00 [28.00, 32.00]	31.00 [27.75, 34.00]	**0.003**
Resilience at Work score (/120)		88.00 [76.00, 96.00]	89.00 [84.00, 95.00]	92.00 [82.00, 97.00]	**0.045**
Wong and Law Emotional Intelligence Score (/112)		90.00 [79.00, 96.00]	93.00 [88.00, 99.00]	95.50 [88.75, 101.00]	**<0.001**
Income meets financial needs.		3.00 [2.00, 4.00]	3.00 [2.00, 3.00]	3.00 [2.00, 3.00]	**<0.001**
Change in household income	Same	140 (39.2%)	34 (17.1%)	20 (18.5%)	**<0.001**
	Decreased	79 (22.1%)	84 (42.2%)	18 (16.7%)	
	Increased	138 (38.7%)	81 (40.7%)	70 (64.8%)	
Received COVID-19-related supplemental income	No	143 (40.1%)	62 (31.2%)	24 (22.2%)	**0.001**
	Yes	214 (59.9%)	137 (68.8%)	84 (77.8%)	
Years in home and community care		5.00 [3.00, 10.00]	7.00 [4.00, 10.00]	5.00 [4.00, 9.00]	**<0.001**
Weekly hours worked		30.00 [19.00, 37.00]	30.00 [7.50, 36.50]	30.00 [26.00, 36.00]	**0.002**
Work in other sectors	No	118 (33.1%)	42 (21.1%)	16 (14.8%)	**<0.001**
	Yes	239 (66.9%)	157 (78.9%)	92 (85.2%)	
Employment status	Full-time in 1 or more roles	204 (57.1%)	160 (80.4%)	89 (82.4%)	**<0.001**
	Part-time	153 (42.9%)	39 (19.6%)	19 (17.6%)	
Worked in new supervisor role	No	192 (53.8%)	44 (22.1%)	24 (22.2%)	**<0.001**
	Yes	139 (38.9%)	135 (67.8%)	75 (69.4%)	
	Already worked as supervisor	26 (7.3%)	20 (10.1%)	9 (8.3%)	
Ontario Health Region	West	131 (36.7%)	72 (36.2%)	28 (25.9%)	**0.008**
	Central	106 (29.7%)	73 (36.7%)	38 (35.2%)	
	Toronto	30 (8.4%)	25 (12.6%)	20 (18.5%)	
	East	65 (18.2%)	23 (11.6%)	17 (15.7%)	
	North (East and West)	25 (7.0%)	6 (3.0%)	5 (4.6%)	
**(B)**					
**Variable**	**Description**	**Stay**	**Temporary Leave**	**Exit**	***p*** **-Value**
		**n (%) or ** **Median [IQR]**	**n (%) or ** **Median [IQR]**	**n (%) or ** **Median [IQR]**	
n		357 (53.8%)	199 (30.0%)	108 (16.3%)	
Increase in caseload		3.00 [3.00, 4.00]	4.00 [3.00, 4.00]	3.00 [2.00, 4.00]	**<0.001**
Increase in travel		3.00 [2.00, 4.00]	3.00 [3.00, 4.00]	3.00 [2.00, 4.00]	**<0.001**
Increase in virtual appointments		3.00 [2.00, 4.00]	3.00 [2.00, 4.00]	3.00 [2.00, 4.00]	0.240
Increase in non-client hours		3.00 [2.00, 3.00]	3.00 [2.00, 4.00]	3.00 [2.00, 3.00]	**0.007**
Increase in referrals		3.00 [2.00, 4.00]	3.00 [3.00, 4.00]	3.50 [3.00, 4.00]	**0.047**
Increase in case severity		3.00 [2.00, 4.00]	3.00 [3.00, 4.00]	3.00 [2.00, 4.00]	**0.002**
Increase in caseload variability		3.00 [3.00, 4.00]	3.00 [3.00, 4.00]	3.00 [3.00, 4.00]	0.568
Clear communication		3.50 [3.00, 4.00]	3.50 [3.00, 4.00]	3.25 [3.00, 3.50]	**0.002**
Concise communication		3.00 [2.50, 3.50]	3.00 [3.00, 3.50]	3.00 [2.50, 3.50]	**<0.001**
Relevant communication		3.50 [3.00, 4.00]	3.50 [3.00, 4.00]	3.50 [3.00, 4.00]	0.777
Timely communication		3.50 [3.00, 3.50]	3.00 [2.50, 3.50]	3.00 [2.50, 3.50]	**0.050**
Satisfaction with income		3.00 [3.00, 4.00]	4.00 [3.00, 4.00]	3.00 [3.00, 4.00]	**0.046**
Satisfaction with professional development opportunities		3.00 [3.00, 4.00]	3.00 [3.00, 4.00]	3.00 [3.00, 4.00]	0.974
Satisfaction with education opportunities		3.00 [3.00, 4.00]	3.00 [2.00, 4.00]	3.00 [2.00, 4.00]	0.169
Satisfaction with benefits		3.00 [2.00, 4.00]	3.00 [2.50, 4.00]	3.00 [3.00, 4.00]	0.568
Satisfaction with EAP		3.00 [3.00, 4.00]	3.00 [3.00, 4.00]	3.00 [3.00, 4.00]	0.727
Satisfaction with Rewards/Recognition		3.00 [3.00, 4.00]	3.00 [3.00, 4.00]	3.00 [3.00, 4.00]	0.960
Satisfaction with IPC training		3.00 [3.00, 4.00]	3.00 [2.00, 4.00]	3.00 [2.00, 4.00]	0.574
Satisfaction with access to COVID-19 information from employer		3.00 [2.00, 4.00]	3.00 [2.00, 4.00]	3.00 [2.00, 4.00]	0.058
Satisfaction with access to COVID-19 information from CNO		3.00 [3.00, 4.00]	3.00 [3.00, 4.00]	3.00 [2.75, 4.00]	0.577
Satisfaction with access to IPAC support		3.00 [2.00, 4.00]	3.00 [3.00, 4.00]	3.00 [2.00, 4.00]	**0.010**
Satisfaction with supervisor communication about COVID-19		3.00 [2.00, 4.00]	3.00 [3.00, 4.00]	3.00 [2.00, 4.00]	0.625
Satisfaction with employer communication about COVID-19 protocols		3.00 [2.00, 4.00]	3.00 [3.00, 4.00]	3.00 [3.00, 4.00]	**0.039**
Satisfaction with connection with supervisor		3.00 [3.00, 4.00]	3.00 [3.00, 4.00]	3.00 [3.00, 4.00]	0.085
Satisfaction with connection with employer		3.00 [3.00, 4.00]	4.00 [3.00, 4.00]	3.00 [3.00, 4.00]	0.085
Satisfaction with PPE access		3.00 [3.00, 4.00]	4.00 [3.00, 4.00]	3.00 [2.00, 4.00]	**<0.001**
Satisfaction with PPE quality		3.00 [2.00, 4.00]	3.00 [3.00, 4.00]	3.00 [3.00, 4.00]	**0.003**
Feel part of a team		3.00 [3.00, 4.00]	4.00 [3.00, 4.00]	3.00 [3.00, 4.00]	0.072
Have opportunities to connect with supervisor		3.00 [2.00, 3.00]	3.00 [3.00, 4.00]	3.00 [2.00, 4.00]	**0.003**
Have opportunities to connect with colleagues		3.00 [3.00, 4.00]	3.00 [2.00, 4.00]	3.00 [2.00, 4.00]	**0.045**
Feel employer cares about wellbeing		3.00 [3.00, 4.00]	3.00 [3.00, 4.00]	3.00 [3.00, 4.00]	0.701
Pandemic impact on household responsibilities		3.00 [2.00, 4.00]	3.00 [3.00, 4.00]	3.00 [3.00, 4.00]	0.744
Pandemic impact on parenting/caregiving responsibilities		2.50 [2.00, 3.50]	3.00 [2.50, 3.50]	3.00 [2.00, 3.00]	**0.033**
Pandemic impact on friend relationships		3.00 [1.00, 4.00]	3.00 [2.00, 4.00]	3.00 [2.00, 4.00]	**<0.001**
Pandemic impact on family relationships		3.00 [2.00, 4.00]	3.00 [3.00, 4.00]	3.00 [3.00, 4.00]	0.125
Pandemic impact on professional network		3.00 [2.00, 4.00]	3.00 [2.50, 4.00]	3.00 [2.00, 4.00]	**<0.001**
Pandemic impact on personal hygiene		3.00 [2.00, 4.00]	3.00 [3.00, 4.00]	3.00 [3.00, 4.00]	0.076

Note: IQR = Interquartile Range. bolded items indicate significant *p* values.

**Table 3:** Multinomial logistic regression model associations with decision to work in HCC.

Variable	Description	Temporary Leave (Reference: Stay)				Exit (Reference: Stay)			
		Est.	SE	*p*-Value	RRR	Est.	SE	*p*-Value	RRR
Gender	Women/ Gender-diverse/ Prefer not to say	Reference							
	Man	0.32	0.23	0.161	1.38 (0.88, 2.16)	0.41	0.29	0.152	1.51 (0.86, 2.66)
Ethnicity	White	Reference							
	Black	−0.35	0.36	0.329	0.70 (0.35, 1.43)	−0.92	0.46	**0.047**	**0.40 (0.16, 0.99)**
	East/Southeast Asian	0.40	0.54	0.463	1.49 (0.51, 4.32)	0.57	0.61	0.343	1.78 (0.54, 5.82)
	Other identities	0.81	0.38	**0.032**	**2.25 (1.07, 4.70)**	−0.03	0.51	0.955	0.97 (0.35, 2.66)
	South Asian	0.60	0.40	0.138	1.82 (0.83, 4.02)	1.06	0.48	**0.025**	**2.90 (1.14, 7.35)**
Age		0.02	0.02	0.206	1.03 (0.99, 1.07)	−0.02	0.02	0.399	0.98 (0.94, 1.03)
Marital status	Married/Common Law	Reference							
	Separated/Divorced	0.27	0.35	0.439	1.31 (0.66, 2.60)	1.12	0.41	**0.007**	**3.08 (1.37, 6.93)**
	Single/Widowed	−0.54	0.36	0.131	0.58 (0.29, 1.17)	-0.66	0.50	0.186	0.52 (0.20, 1.37)
Past health status		−0.17	0.14	0.236	0.85 (0.64, 1.12)	−0.44	0.18	**0.013**	**0.64 (0.45, 0.91)**
Test positive for COVID-19	No	Reference							
	Yes	1.49	0.29	**<0.001**	**4.43 (2.53, 7.77)**	1.27	0.34	**<0.001**	**3.55 (1.83, 6.89)**
Wong and Law Emotional Intelligence Score		0.04	0.01	**0.010**	**1.04 (1.01, 1.07)**	0.09	0.02	**<0.001**	**1.10 (1.06, 1.14)**
Income meets financial needs		−0.16	0.14	0.261	0.85 (0.64, 1.13)	0.10	0.17	0.579	1.10 (0.78, 1.55)
Change in household income	Same	Reference							
	Decreased	0.90	0.34	**0.008**	**2.45 (1.27, 4.74)**	0.45	0.50	0.369	1.56 (0.59, 4.12)
	Increased	0.89	0.33	**0.007**	**2.44 (1.27, 4.69)**	1.51	0.41	**<0.001**	**4.52 (2.04, 10.02)**
Received COVID-19-related supplemental income	No	Reference							
	Yes	0.63	0.25	**0.012**	**1.88 (1.15, 3.08)**	0.86	0.33	**0.010**	**2.35 (1.23, 4.51)**
Employment status	Full-time in 1 or more roles	Reference							
	Part-time	−0.45	0.28	0.107	0.64 (0.37, 1.10)	−0.34	0.36	0.342	0.71 (0.36, 1.43)
Years in home and community care		−0.12	0.04	**0.001**	**0.89 (0.83, 0.96)**	−0.13	0.05	**0.007**	**0.88 (0.80, 0.97)**
Weekly hours worked		−0.03	0.01	**0.033**	**0.97 (0.95, 0.998)**	0.02	0.02	0.280	1.02 (0.99, 1.05)
Worked in new supervisor role	No	Reference							
	Already worked as supervisor	1.03	0.42	**0.014**	**2.80 (1.23, 6.36)**	0.03	0.56	0.954	1.03 (0.35, 3.09)
	Yes	0.42	0.31	0.175	1.52 (0.83, 2.79)	0.44	0.38	0.253	1.55 (0.73, 3.29)
Work in other sectors	No	Reference							
	Yes	−0.33	0.29	0.265	0.72 (0.41, 1.28)	0.49	0.38	0.206	1.63 (0.77, 3.45)
Ontario Health Region	West	Reference							
	Central	0.36	0.27	0.183	1.44 (0.84, 2.45)	0.33	0.36	0.355	1.39 (0.69, 2.80)
	East	−0.02	0.35	0.948	0.98 (0.49, 1.94)	0.61	0.43	0.154	1.84 (0.80, 4.30)
	North [East and West]	−0.39	0.55	0.478	0.68 (0.23, 1.98)	0.05	0.66	0.943	1.05 (0.29, 3.85)
	Toronto	0.81	0.39	**0.035**	**2.26 (1.06, 4.81)**	1.53	0.46	**0.001**	**4.63 (1.87, 11.45)**
Increase in caseload		−0.03	0.10	0.752	0.97 (0.79, 1.19)	−0.32	0.13	**0.013**	**0.73 (0.57, 0.94)**
Increase in caseload variability		−0.01	0.12	0.963	0.99 (0.79,1.26)	−0.26	0.14	0.060	0.77 (0.58,1.01)
Concise communication		0.44	0.19	**0.018**	**1.56 (1.08, 2.24)**	−0.56	0.22	**0.012**	**0.57 (0.37, 0.89)**
Relevant communication		−0.60	0.20	**0.002**	**0.55 (0.37, 0.80)**	−0.10	0.24	0.668	0.90 (0.56, 1.45)
Timely communication		−0.30	0.17	0.078	0.74 (0.53, 1.03)	−0.49	0.21	**0.019**	**0.61 (0.40, 0.92)**
Satisfaction with employer communication about COVID-19 protocols		0.26	0.12	**0.034**	**1.30 (1.02, 1.66)**	0.39	0.15	**0.009**	**1.48 (1.10, 1.99)**
Satisfaction with supervisor communication about COVID-19		−0.11	0.13	0.401	0.90 (0.69, 1.16)	−0.36	0.15	**0.019**	**0.70 (0.52, 0.94)**
Satisfaction with benefits		0.43	0.13	**0.001**	**1.53 (1.18, 1.99)**	0.25	0.16	0.131	1.28 (0.93, 1.77)
Satisfaction with PPE access		0.05	0.13	0.685	1.05 (0.82, 1.36)	−0.29	0.15	0.062	0.75 (0.56, 1.02)
Satisfaction with PPE quality		0.21	0.14	0.119	1.24 (0.95, 1.62)	0.22	0.16	0.179	1.25 (0.90, 1.72)
Have opportunities to connect with colleagues		0.00	0.13	0.982	1.00 (0.77, 1.29)	−0.36	0.15	**0.021**	**0.70 (0.52, 0.95)**
Pandemic impact on caregiving responsibilities		0.01	0.14	0.961	1.01 (0.76, 1.34)	−0.29	0.18	0.110	0.75 (0.52, 1.07)
Pandemic impact on professional network		0.04	0.11	0.760	1.04 (0.83, 1.30)	0.44	0.14	**0.002**	**1.56 (1.18, 2.06)**
Pandemic impact on friend relationships		0.22	0.11	**0.035**	**1.25 (1.02, 1.54)**	0.10	0.13	0.442	1.11 (0.85, 1.44)

Note: Est = Estimate, SE = Standard Error, and RRR = Relative Risk Ratio. bolded items indicate significant *p* values.

## Data Availability

The original contributions presented in the study are included in the article; further inquiries can be directed to the corresponding author.
